# SATB1-Mediated Upregulation of the Oncogenic Receptor Tyrosine Kinase HER3 Antagonizes MET Inhibition in Gastric Cancer Cells

**DOI:** 10.3390/ijms22010082

**Published:** 2020-12-23

**Authors:** Robert Jenke, Miriam Holzhäuser-Rein, Stefanie Mueller-Wilke, Florian Lordick, Achim Aigner, Thomas Büch

**Affiliations:** 1 University Cancer Center Leipzig (UCCL), University Hospital Leipzig, D-04103 Leipzig, Germany; robert.jenke@medizin.uni-leipzig.de (R.J.); mr41refa@studserv.uni-leipzig.de (M.H.-R.); Stefanie.Mueller3@medizin.uni-leipzig.de (S.M.-W.); florian.lordick@medizin.uni-leipzig.de (F.L.); 2 Clinical Pharmacology, Rudolf-Boehm-Institute for Pharmacology and Toxicology, Medical Faculty, Leipzig University, D-04107 Leipzig, Germany; thomas.buech@medizin.uni-leipzig.de

**Keywords:** gastric cancer, HER3, heregulin, MET, SATB1

## Abstract

MET-amplified gastric cancer cells are extremely sensitive to MET inhibition in vitro, whereas clinical efficacy of MET inhibitors is disappointing. The compensatory activation of other oncogenic growth factor receptors may serve as an underlying mechanism of resistance. In this study, we analyzed the role of HER receptors, in particular HER3 and its ligand heregulin, in this respect. This also included the chromatin-organizer protein SATB1, as an established regulator of HER expression in other tumor entities. In a panel of MET-amplified gastric carcinoma cell lines, cell growth under anchorage-dependent and independent conditions was studied upon inhibitor treatment or siRNA-mediated knockdown. Expression analyses were performed using RT-qPCR, FACS, and immunoblots. Signal transduction was monitored via antibody arrays and immunoblots. As expected, MET inhibition led to a growth arrest and inhibition of MAPK signaling. Strikingly, however, this was accompanied by a rapid and profound upregulation of the oncogenic receptor HER3. This finding was determined as functionally relevant, since HER3 activation by HRG led to partial MET inhibitor resistance, and MAPK/Akt signaling was even found enhanced upon HRG+MET inhibitor treatment compared to HRG alone. SATB1 was identified as mediator of HER3 upregulation. Concomitantly, SATB1 knockdown prevented upregulation of HER3, thus abrogating the HRG-promoted rescue from MET inhibition. Taken together, our results introduce the combined HER3/MET inhibition as strategy to overcome resistance towards MET inhibitors.

## 1. Introduction

Gastric cancer is one of the most common cancer types [1,2]. Worldwide, it represents the second or third most common cause of cancer-related deaths [3,4], and the lifetime risk of developing gastric cancer is about one case per 100 persons. Until now, complete surgical resection of the tumor is a prerequisite for curative treatment [5,6]. However, gastric cancer is often advanced and inoperable at the time point of diagnosis, and conventional cytoreductive chemotherapy is of rather limited efficacy. Thus, there is a desperate need for novel systemic treatment approaches to improve prognosis, especially in metastatic gastric cancer [7]. Recently, targeted therapies against oncogenic receptor tyrosine kinases (RTKs), e.g., FGFR, HER1, HER2, HER3, or MET, have been tested in patients with gastric cancer [8]. Despite promising findings in cell culture, the clinical efficacy of these novel therapeutics has been rather limited in most cases, with the partial exception of HER2 inhibition, showing a statistically significant albeit small survival advantage in a subset of patients with HER2 overexpression [9].

Discrepancies between preclinical data and the clinical reality are particularly striking in the case of the inhibition of the HGF/MET axis in gastric cancer cells harboring a MET amplification, which occurs in 3–7% of gastric tumors [10,11]. In these cells, marked anti-proliferative effects are observed in vitro after inhibition of MET; however, until now clinical trials with HGF or MET inhibitors have not produced any breakthrough [12]. The difficulties of translating positive preclinical data into favorable clinical outcomes can be attributed in part to the problem of identifying the correct subgroup of patients suitable for a specific molecularly defined targeted therapy. While it appears reasonable to pre-select patients with tumors showing high expression levels of the respective target molecule, it should be noted that the overexpression of a given oncogene does not necessarily translate into high sensitivity of tumor cells towards its inhibition. This indicates that expression levels may be a poor predictor of therapy response, with the potential redundancy of oncogenic signaling pathways in tumors being one explanation for this discrepancy. Indeed, upon inhibition of a distinct critical pathway, for example a specific RTK, the activation of other signaling molecules can compensate for its reduced function [13,14]. 

It has been proposed that members of the HER family of RTKs, especially HER2 or HER3, could compensate for a reduced MET function, thus contributing to tumor resistance against MET inhibitors. In fact, stimulation of MET-amplified gastric cancer cells with the HER agonist heregulin (HRG) could ameliorate the cytotoxic effects of MET inhibitors [15,16,17]. From these findings, the questions arise (1) whether the HRG rescue effect is relevant for all MET-amplified gastric cancer cells, (2) if alterations in HER receptor expression and/or signaling are observed upon MET inhibition, (3) whether HRG elicits its positive effects via HER1/HER3 or HER2/HER3-promoted survival signaling in this context, and (4) which other molecules are involved in MET resistance. The chromatin organizer protein SATB1 has been shown to be upregulated in many solid tumors and, as proto-oncogene, to affect the expression of many tumor-relevant gene products including HER receptors [18,19,20]. Thus, we hypothesized that SATB1 could also be involved in HER-dependent resistance mechanisms upon MET inhibition in gastric cancer cells.

In this study, we address the functional relevance of HER receptors, and in particular of HER3, in MET-amplified gastric cancer cell lines. This also includes the role of SATB1 in this process. We show a rapid and substantial increase in HER3 expression upon inhibiting MET, which is mediated by SATB1 and leads to an even enhanced heregulin (HRG)/HER3 signaling. This establishes the role of HRG/HER3 signaling in mediating resistance of gastric cancer cells towards MET inhibition. Thus, our findings provide an avenue towards increasing the efficacy of MET-directed therapeutic interventions. 

## 2. Results

### 2.1. MET-Amplified Gastric Cancer Cells Are Highly Sensitive to MET Inhibition or siRNA-Mediated MET Knockdown

**Figure 1:**
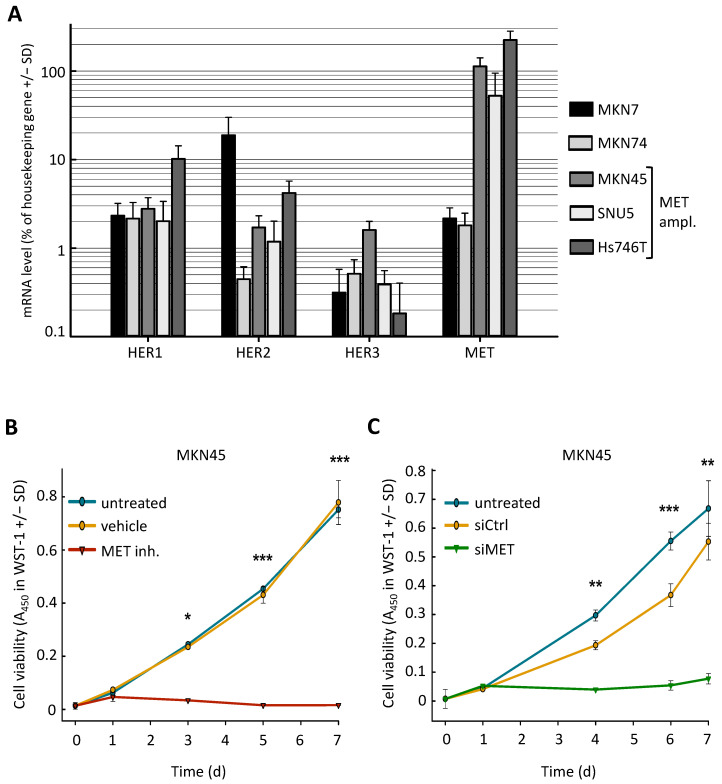
(**A**) Expression levels of MET and HER receptor mRNA in different gastric carcinoma cell lines. mRNA expression was determined using quantitative RT-PCR using RPLP0 as housekeeping gene. (**B**,**C**) Effects of MET inhibition via small molecule inhibitor or siRNA on tumor cell proliferation in MET-amplified MKN45 cells. Using WST-1 reagent cell proliferation was monitored on day 0 (prior to treatment) and day 3, 5, and 7. Upon treatment with 0.2 µM MET inhibitor PF04217903 (**B**) or siRNA-mediated MET knockdown (**C**), profound inhibition was observed. Level of significance: *, *p* < 0.05; **, *p* < 0.01, and ***, *p* < 0.001.

To investigate the role of HER receptors in resistance of gastric cancer cells against MET inhibitors, we used a panel of five gastric cancer cell lines, three of which (MKN45, Hs746T, and SNU5) were described as MET-amplified and sensitive to MET inhibition, and two (MKN7 and MKN74) are not MET-amplified. Expression analyses on the mRNA level confirmed the exceptionally high MET expression in the three MET-amplified cell lines (Figure 1A). MET-amplified cells showed high sensitivity towards MET inhibition via the specific inhibitor PF04217903 (0.2 µM) (Figure 1B, Supplementary Materials Figure S1A,B) or siRNA-mediated downregulation of MET (Figure 1C). In contrast, no anti-proliferative effects were observed in cell lines MKN74 or MKN7 without MET amplification, even for MET inhibitor concentrations of up to 2 µM or following siRNA-mediated MET knockdown (Supplementary Materials Figure S2A–D).

### 2.2. Downregulation or Inhibition of MET Leads to Upregulation of HER3

**Figure 2:**
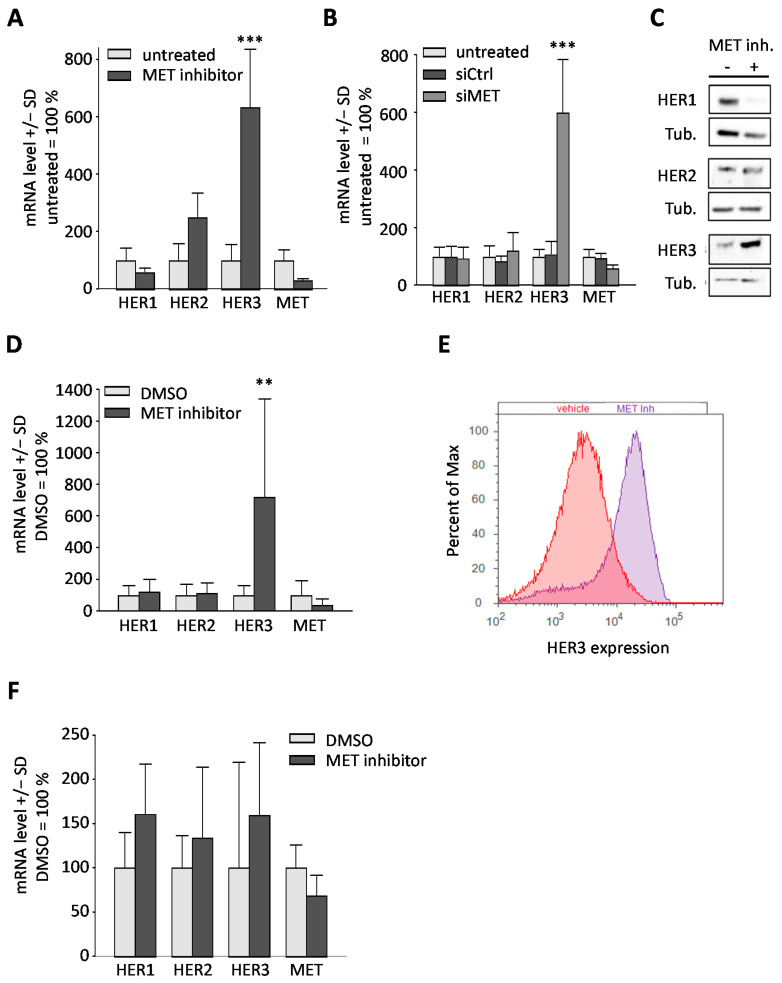
Inhibition of MET leads to HER3 receptor upregulation in MET-amplified MKN45 and SNU5 cells, but not in Hs746T cells. (**A**) After treatment of MKN45 cells, with MET inhibitor PF04217903 (0.2 µM for 48 h) a pronounced upregulation of HER3 was traceable on mRNA level. (**B**) Transfection of MKN45 cells with specific siRNA against MET for 48 h yielded similar HER3 upregulation results. (**C**) Accordingly, 48 h treatment of MKN45 cells with 0.2 µM of PF04217903 also led to upregulation of HER3 on protein level, whereas differential effects occurred for HER1 and HER2. (**D**) In SNU5 cells, treatment with 0.2 µM PF04217903 also showed marked HER3 upregulation on mRNA level. (**E**) Moreover, a shift in expression of HER3 protein level was observed after 0.2 µM PF04217903 treatment (48 h). (**F**) Contrastingly, no HER3 upregulation was traceable in Hs746T cells under these conditions. Level of significance: **, *p* < 0.01, and ***, *p* < 0.001.

It has been described previously in non-gastric cancer cell lines that resistance of HER receptor overexpressing cells towards inhibition or knockdown can be attributed to the adaptive activation of other HER family members ([13,14] for review). Thus, we next asked the question whether the targeting of MET, despite its profound cell-inhibitory effects, may lead to similar alterations. Of note, a very strong > 6-fold upregulation of HER3 was detected in MKN45 cells on the mRNA (Figure 2A,B) and protein level (Figure 2C). Western blot data were also confirmed by flow cytometry (Supplementary Materials Figure S3). Since this method is very quantitative and also allows for specifically monitoring cell surface levels, we sticked to flow cytometry for measuring HER3 protein in subsequent experiments. This HER3 upregulation was independent of whether MET inhibition was achieved by siRNA-mediated knockdown or using the inhibitor PF04217903. The same increase in HER3 levels was observed in SNU5 cells on mRNA (Figure 2D) and protein level (Figure 2E). In contrast, in Hs746T cells a less pronounced ~1.5 increase in HER3 was observed, but in this cell line, it was accompanied by a concomitant induction of HER1 and HER2 in the same range (Figure 2F).

Additionally, various responses were noted with regard to HER1 and HER2 levels: in MKN45 cells, HER1 mRNA was slightly reduced upon MET inhibitor treatment (Figure 2A), but not after RNAi-mediated knockdown of MET (Figure 2B). Of note, these HER1 effects upon MET inhibitor treatment were also discernible on protein level (Figure 2C). In contrast, in SNU5 cells no major effects were found (Figure 2D), and in Hs746T cells, even a minor HER1 induction occurred (Figure 2F). Regarding HER2 expression, a strong mRNA induction was discernible in MKN45 cells (Figure 2A), which was, however, not seen on protein levels (Figure 2C) and may be, therefore, of minor relevance. For the other cell lines, only weak effects on HER2 were found (Figure 2D,F). Additionally, the determination of mRNA levels also revealed that treatment of cells with the MET inhibitor led to a marked reduction in MET after 48 h, indicating an inhibitory effect of PF04217903 on the transcription of its target (Figure 2A,D,F). Taken together, this identifies HER3 as a candidate oncogene for mediating resistance towards MET inhibition. 

### 2.3. Anti-Proliferative Effects of MET Inhibition Are Partially Abolished by Treatment with HER3 Activator Heregulin

**Figure 3:**
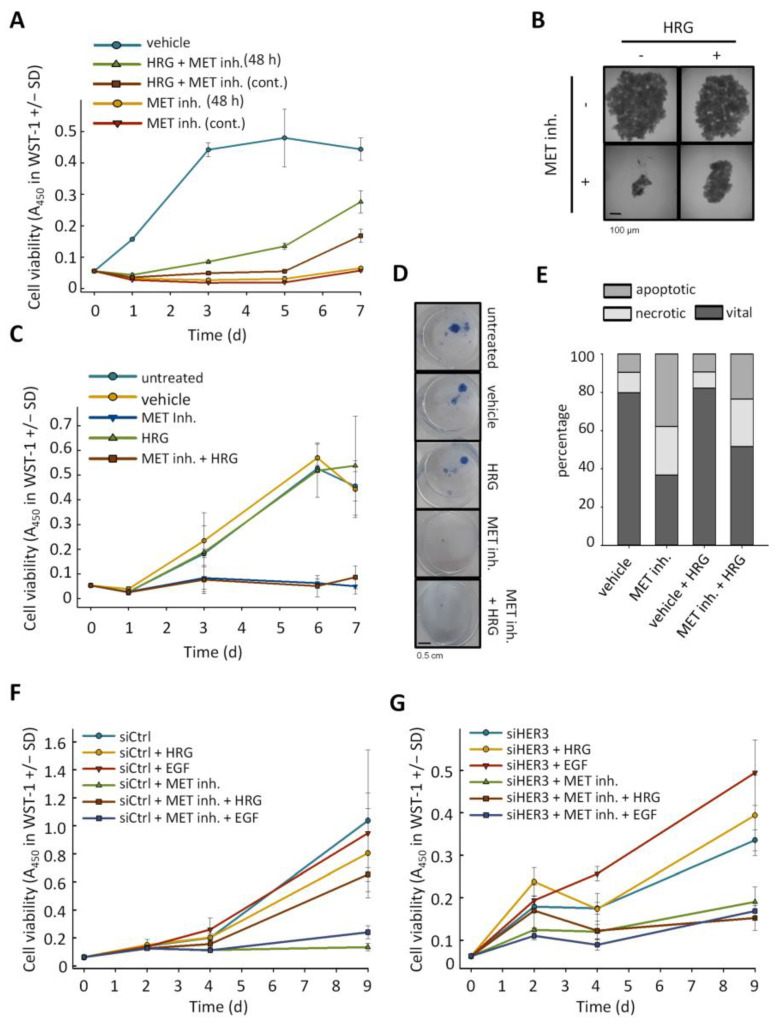
(**A**) In WST-1 assay, impaired MKN45 cell proliferation (vs. vehicle DMSO) upon MET inhibition is partially rescued by treatment with 20 ng/mL of heregulin (HRG); (cont.): continuous inhibitor (0.2 µM PF04217903) exposure, (48 h): removal of MET inhibitor after 48 h. (**B**) HRG (20 ng/mL) also partially reversed antiproliferative effects of MET inhibition in MKN45 cells on spheroid formation. In contrast, the severe effects upon MET inhibition could not be reversed by HRG in Hs746T cells as shown (**C**) in WST-1 assay or (**D**) spheroid outgrow formation; of note, these cells did not show compensatory HER3 upregulation upon MET inhibition, as has been shown in the previous Figure 2F. (**E**) Addition of HRG (20 ng/mL) also reduced the number of apoptotic cells in SNU5 cells treated with 0.2 µM PF04217903. HER3 displays the crucial factor mediating resistance against MET inhibition, as (**F**) the HER1 ligand EGF (50 ng/mL) could not reverse the antiproliferative effects of PF04217903 in contrast to HRG treatment, and (**G**) HER3 knockdown abrogated the HRG-induced rescue effects.

The interplay between MET inhibition and alterations in HER receptor expression levels suggested the possibility that the very profound anti-proliferative effects of the MET inhibitor may be counteracted by HER3 activation in the presence HER receptor ligands. Indeed, addition of heregulin (HRG) in the physiological concentration of 20 ng/mL to the culture media led to a partial rescue of MET inhibitor-mediated (0.2 µM of PF04217903) arrest in proliferation in MKN45 cells. This was even true in the constant presence of the inhibitor and thus under conditions of sustained MET inhibition (Figure 3A). In the absence of HRG, earlier removal of the inhibitor after 48 h did not lead to reduced inhibition of cell proliferation over time but resulted in a further enhanced HRG-mediated rescue effect (Figure 3A). Thus, albeit HRG could not fully compensate for the MET inhibitor effects, a major recovery of cell proliferation was observed. Likewise, in colony formation assays MET inhibitor (0.2 µM of PF04217903 for 48 h) severely impaired MKN45 colony formation, an effect that was reversed by HRG treatment (Supplementary Materials Figure S4). This protective effect was also seen in a spheroid growth assay, where the MET inhibitor alone almost completely abrogated spheroid growth, whereas in the presence of HRG, the three-dimensional growth was partially retained with spheroid sizes reaching ~30% of the control cells (Figure 3B). In SNU5 cells, comparable results were obtained (data not shown). In contrast, in Hs476T cells lacking the very profound HER3 induction upon MET inhibition (see Figure 2D), HRG treatment could not antagonize the growth inhibition of the MET inhibitor in WST-1 assays (Figure 3C) or spheroid outgrowth assays (Figure 3D). This identifies HER3 upregulation, in combination with the presence of its ligand HRG, as a mediator of resistance towards MET inhibition. 

When analyzing the percentages of viable and dead cells in a live/dead cell assay, a substantial > 4-fold increase in apoptotic cells upon exposure to the MET inhibitor was observed. Again, however, this was markedly reduced in the combined treatment scenario with MET inhibitor plus HRG, indicating the pro-survival effects of HER3/HRG signaling under MET inhibition (Figure 3E). 

The functional relevance of HER3 was further explored by siRNA-mediated parallel HER3 knockdown. In negative control transfected cells and in the absence of MET inhibitor, no further stimulation of cell proliferation was obtained upon addition of HRG or the HER1 ligand EGF (Figure 3F, left). In contrast, the proliferation arrest exerted by the MET inhibitor could again be rescued by >50% upon addition of HRG, while treatment with the HER1 ligand EGF was without effect (Figure 3F, left). This identifies HER3 rather than HER1 as relevant in this context. Upon siRNA-mediated transient HER3 knockdown, a marked reduction in cell proliferation was seen (Figure 3G; note the y-axis scale different to Figure 3F). This was further augmented by parallel treatment with the MET inhibitor. Notably, the HER3 knockdown abolished the recovery of cell proliferation upon addition of HRG (Figure 3F, right), indicating that the HRG-mediated rescue described above is indeed dependent on HER3. 

### 2.4. Cellular Effects Are Mediated by Alterations in MAPK Signaling

**Figure 4:**
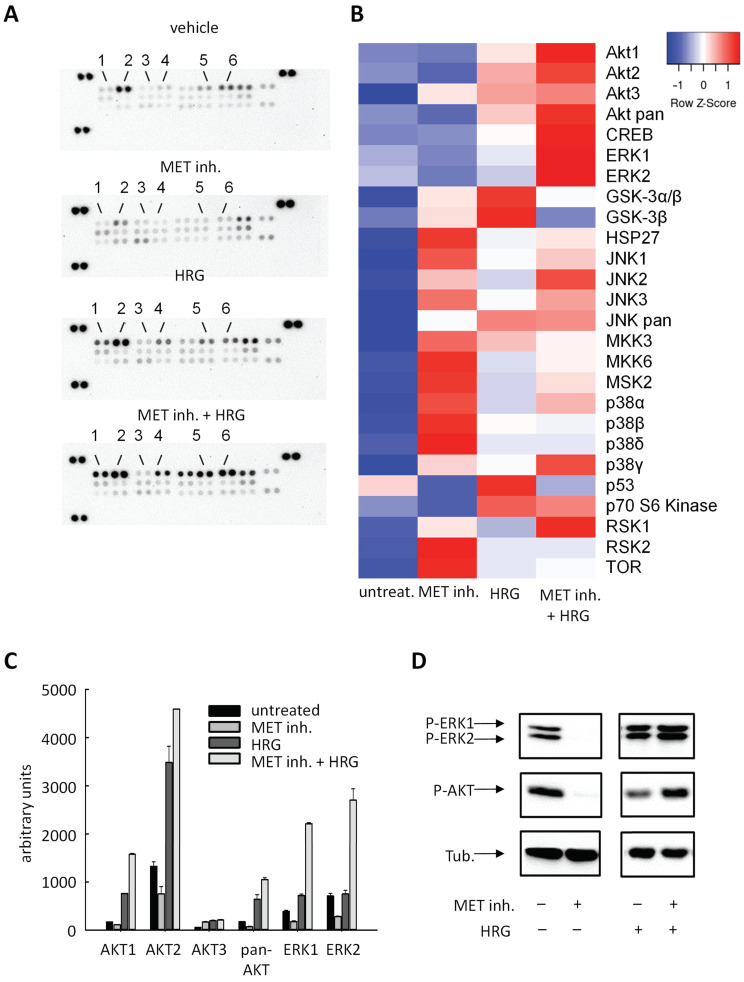
(**A**) Phospho-antibody arrays to elucidate downstream signal transduction effects of MET inhibition, heregulin stimulation, and the combination of both as compared to untreated (upper panel). 1, Akt1; 2, Akt2; 3, Akt3; 4, pan Akt; 5, ERK1; 6, ERK2. For the definition of all spots, see Supplementary Materials Figure S5. Cells were serum-starved for 18 h and subsequently treated with vehicle DMSO or inhibitor/HRG for 24 h. Interestingly, MET inhibition followed by HER3 stimulation via HRG showed the strongest phosphorylation levels. (**B**) Quantification of signal intensities from antibody arrays. The heat map depicts alterations upon treatment as indicated towards lower (blue) or higher signals (red). (**C**) Bar diagram showing the intensities of signaling molecules with most profound alterations. (**D**) Confirmation of alterations in ERK1/2 and Akt phosphorylation by Western blotting. Again, cells were serum-starved for 18 h and treated as indicated for 24 h.

To further characterize the pathways involved in the cellular effects of MET inhibition and HRG stimulation/rescue, phospho-antibody arrays were performed for analyzing changes in MAPK activities (Figure 4A,B, Figure S5). Upon addition of the MET inhibitor to MKN45 cells, reduced phosphorylation of Akt (especially Akt2) and of ERK1/2 was observed (Figure 4A). In contrast, HRG stimulation of the cells led to further enhancement of Akt signaling, with little effects on ERK1/2 phosphorylation. Notably, the inhibitory effects of the MET inhibitor on phosphorylation/activation were, except for Akt3, reversed upon HRG addition (Figure 4A, lower panel). The heat map analysis confirmed Akt and ERK to be most profoundly affected. In fact, the quantitation of the signals revealed an even increase in Akt signaling upon combined MET inhibitor + HRG treatment and very profound ~3–4-fold higher ERK1/2 phosphorylation (Figure 4C). This further increase in Akt and ERK1/2 signaling to values above those obtained by HRG stimulation alone without MET inhibitor was also confirmed in independent Western blot experiments (Figure 4D, right panel). The profoundly increased p-ERK1/2 and p-Akt levels in MET + HRG treated cells over HRG single treatment can be explained by the upregulation of HER3 (and perhaps HER2) described above. 

### 2.5. Upregulation of HER3 upon MET Inhibition Is Dependent on PKC and SATB1

We further analyzed the underlying molecular mechanism of this counter-upregulation. We did not find any evidence that the blockade of MAPK or AKT signaling induced by MET inhibition was involved in HER3 regulation, since the inhibition of MAPK by the MAPKK inhibitor PD98059 (10 µM for 48 h) or the blocking of AKT signaling via PI3K inhibition with LY294002 (10 µM for 48 h) did not reproduce the effects of MET inhibition on HER3 levels (Supplementary Materials Figure S6). 

**Figure 5:**
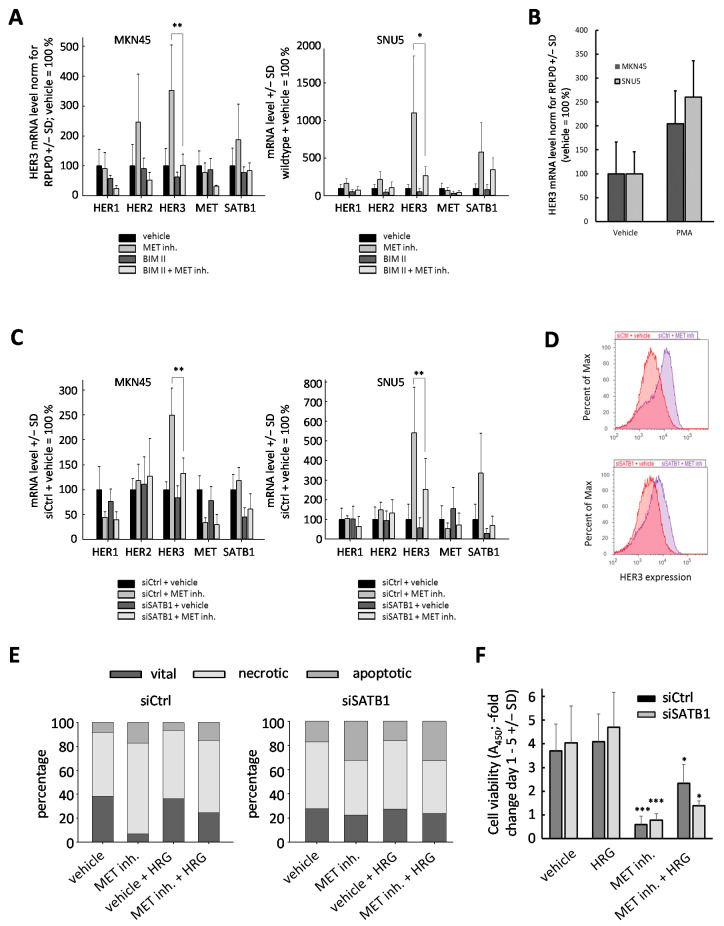
(**A**) MKN45 cells (left) and SNU5 cells (right) were pretreated with PKC inhibitor BIM II (10 µM) for 24 h; afterwards, cells were treated with MET inhibitor PF04217903 (0.2 µM for 48 h). (**B**) MKN45 and SNU5 cells were treated for 48 h with the PKC activator PMA (1 µM) before analyzing HER3 mRNA expression. (**C**) MKN45 cells (left) and SNU5 cells (right) were pretreated with SATB1 siRNA for 48 h prior to MET inhibition with 0.2 µM PF04217903 to induce compensatory upregulation effects, as shown previously. siSATB1 significantly reduced the magnitude of observed HER3 mRNA upregulation. (**D**) SNU5 cells also showed diminished HER3 upregulation on the protein level after SATB1 knockdown (48 h after transfection) in comparison with control siRNA. (**E**,**F**) While siSATB1 showed no antiproliferative effect itself on MKN45 cells, the HRG-associated rescue effect was reduced upon SATB1 knockdown as compared to siCtrl. Level of significance: *, *p* < 0.05; **, *p* < 0.01, and ***, *p* < 0.001.

Since PKC is a known MET target, we next tested its role by pretreating MKN45 or SNU5 cells with the PKC inhibitor BIM II (10 µM) for 24 h, prior to the addition of the MET inhibitor PF 04217903 (0.2 µM for 48 h). Under these conditions the upregulation of HER3 was abrogated in MKN45 (Figure 5A, left) and markedly inhibited in SNU5 cells (Figure 5A, right). For HER1 and HER2, only minor effects were discernible. Vice versa, the PKC activator PMA (1 µM, for 48 h) led to a marked upregulation of HER3 in both MKN45 and SNU5 cells (Figure 5B). Of note, we also observed a strong upregulation of the transcriptional HER regulator SATB1 in SNU5, but not in MKN45 cells (Figure 5A). SATB1 has been shown in breast carcinoma to affect the expression of HER receptors [18,19,20] and was found upregulated in gastric cancer [21]. Thus, we next assessed its role in HER3 upregulation. To this end, we employed a specific siRNA, which was described previously to efficiently reduce SATB1 expression [19]. 

Treatment of MKN45 cells with MET inhibitor again led to ~ 50% decreased HER1 mRNA levels, independent of prior transient transfection with SATB1 siRNA or a non-specific negative control siRNA (Figure 5C, left). Slight effects of SATB1 knockdown on basal (i.e., in the absence of MET inhibitor) expression of HER1 and HER3 were observed. In contrast, the marked upregulation of HER3 under MET inhibitor treatment was almost fully abolished upon SATB1 knockdown (Figure 5C, left). The strong dependence of the HER3 counter-upregulation on SATB1 expression thus indicates that it is mediated through SATB1. Similarly, in SNU5 cells, knockdown of SATB1 abrogated the strong HER3 induction upon MET inhibition (Figure 5C, right). Of note, in this cell line, MET inhibition per se again led to a marked upregulation of SATB1 reproducing the data shown in Figure 5A, right, and underlining the potential interplay between MET signaling, SATB1 function, and HER3 expression. Note that in both cell lines MET inhibition again reduced the expression of MET receptor itself (Figure 5C), indicating a yet unknown putative transcriptional inhibitory mechanism on MET activity. This MET downregulation after MET inhibition was not affected by SATB1 knockdown. 

The SATB1-dependent regulation of HER3 expression upon MET inhibition was also found on the protein level. While the MET inhibitor led to a pronounced increase in HER3 expression in control transfected cells (Figure 5D, upper panel), this effect was markedly reduced upon SATB1 knockdown (Figure 5D, lower panel). Addressing the possible consequences of SATB1 affecting HER3 expression, we analyzed cell viabilities. The RNAi-mediated reduction in SATB1 expression did not lead to major alterations of viable cell numbers in untreated or HRG-stimulated cells, or in cells treated with MET inhibitor (Figure 5E). Notably, however, the HRG-mediated partial restoration of cell proliferation under MET inhibition was almost completely abolished upon SATB1 knockdown (Figure 5F, right bars), demonstrating the dependence of this effect on SATB1-mediated HER3 upregulation. 

## 3. Discussion

In the present study, we demonstrate that treatment of MET-amplified gastric cancer cells with a MET inhibitor leads to a SATB1-mediated upregulation of HER3. In the absence of HER3 ligands in cell culture, which are not endogenously produced by the tumor cells, this adaptive and rapid induction of HER3 did not confer resistance towards MET inhibition. In contrast, in the presence of the HER3 ligand heregulin, a scenario, which resembles more closely the in vivo situation in tumors (see below), a partial rescue of the cancer cells from the detrimental effects of MET inhibition was observed. 

It has been shown previously that MET inhibition in monocultured gastric cancer cells with MET amplification exerts dramatic anti-proliferative effects, in parallel with abrogation of ERK and Akt phosphorylation. This can be overcome in part by HER activation [15,16,22]. Heregulin can activate HER3-promoted signaling pathways via HER1/HER3 or HER2/HER3 heterodimers [23]; however, it has not been elucidated so far which of these heterodimers mediate these pro-survival effects in MET addicted gastric cancer cells. We demonstrate that inhibition of HER2 or HER3 via siRNA-mediated knockdown or a small molecule inhibitor abrogated the rescue effect of heregulin, giving proof of the relevance of intact HER2/HER3 signaling. In contrast, we found the treatment of the cells with the HER1 ligand EGF ineffective in mediating any resistance against MET inhibition. This effect cannot be explained by an insufficient dosing of EGF, since EGF treatment led to a comparable reactivation of ERK phosphorylation as did HRG (Supplementary Materials Figure S7). 

The fact that Akt phosphorylation after MET inhibition is more efficiently restored by HRG treatment of gastric cancer cells than by EGF treatment is in line with previous findings [15] and indicates that PI3K-Akt signaling is of particular importance for survival signaling in gastric cancer cells. Somewhat contrasting previous findings that demonstrated the ability of EGF (in a concentration comparable to our study) to confer resistance against MET inhibition as well [15,16] may be attributable to the fact that different MET inhibitors were used. Of note, the inhibitor PHA 665752 used previously at a concentration of 250 nM would also inhibit Ron and at least partially Flk-1 (IC50: 200 nM), whereas PF 04217903 employed here offers greater selectivity towards MET [24]. 

Remarkably, MET inhibition led to a substantial upregulation of HER3, the critical signaling molecule responsible for heregulin-promoted survival. Of note, HER3 has been characterized as a significant factor for tumor progression in gastric cancer and is often upregulated in this tumor entity (see [25] for review). On the transcriptional level, HER3 expression in gastric cancer is critically regulated by the transcription factor EHF and overexpression of EHF leads to increased HER3 levels [26]. With respect to the adaptive response upon MET inhibition observed here, it is noteworthy that PI3K-AKT inhibition, which is a consequence of MET inhibition in MET-amplified gastric cancer cells, can induce HER3 upregulation in other tumor entities via a FOXO-dependent mechanism [27]. However, PI3K-AKT signaling does not seem to play a crucial role in the present context, since PI3K inhibition in gastric cancer cells had no impact on basal HER3 expression or on HER3 induction after MET inhibitor treatment (Supplementary Materials Figure S6).

On the mechanistic level, we identify SATB1 as a mediator of this HER3 upregulation. Concomitantly, SATB1 knockdown abrogated the HRG-promoted rescue of gastric cancer cells after MET inhibition. SATB1 acts as a chromatin organizer, and dependent on the cellular context and on post-translational modifications, SATB1 has been shown to act as a repressor or activator of gene expression [28,29]. SATB1 affects the expression of a large number of oncogenic signaling molecules, and consequently, its function has been studied in several tumor entities [30,31]. While in gastric cancer the role of SATB1 for the regulation of oncogene expression is still elusive, a meta-analysis has revealed that SATB1 expression itself represents a potential marker for unfavorable prognosis, emphasizing its putative relevance in this tumor type [21]. In line with this, SATB1 has been found to increase viability, invasiveness, and chemoresistance of gastric cancer cells and to promote tumor growth in vivo [32,33].

In other tumor entities, a critical role of SATB1 in regulating the expression of receptors of the HER family has been described. More specifically, SATB1 has been shown to be involved in the upregulation of EGFR (HER1), HER2, HER3, and HER4 in breast cancer cells [18]. In contrast, in colorectal cancer cells, SATB1 induced HER3 expression but exerted only mild effects on HER2 and no effect on HER1 expression [19]. In glioma cells, SATB1 was found to even act as a repressor of HER2, since SATB1 knockdown led to an induction of HER2 expression [20]. Collectively, these results support the notion that the role of SATB1 in regulating the expression of different HER receptors strongly depends on the cellular and/or tumor context. Notably, in the gastric cancer cells investigated here, SATB1 knockdown had no impact on the basal expression of any HER receptor; however, the upregulation of HER3 upon MET inhibition was prevented. This further emphasizes the dependence of SATB1 effects on the cellular context.

As mentioned above, in the absence of HER3 ligand MET inhibition was found to decrease ERK and Akt phosphorylation and to completely abrogate cellular proliferation despite of the elevated HER3 expression. This indicates that HER3 overexpression, even after the pronounced increase in HER3 levels upon treatment with MET inhibitors or siRNA, is insufficient to compensate for the blocking of MET-dependent pathways in tumor cells that do not endogenously express heregulin. Thus, 2D cell culture insufficiently reflects the in vivo situation where heregulin expressing and secreting stroma cells are present within the gastric tumor. Notably, heregulin stimulation of MKN45 cells pretreated with MET inhibitor yielded even a higher activation of ERK and Akt signaling than heregulin stimulation of cells with intact MET signaling. Taken together, the induction of HER3 after MET inhibition represents a critical factor in HRG-promoted resistance against MET inhibitors. This can severely impair the effect of MET inhibition, even in tumors with an amplification of MET, and may well explain—at least in part—the poor clinical outcomes of MET inhibitor treatment. The further elucidation of the mechanisms involved in regulation of HER3 expression in gastric cancer could provide the basis for novel strategies improving the efficacy of RTK-targeted therapies. It is noteworthy in this context that heregulin secretion by fibroblasts is a critical homeostatic signal to maintain the integrity of the gastric epithelial lining [34] and that inflammatory processes in the stomach lead to an upregulation of heregulin production of gastric fibroblasts [35]. Concomitantly, we could detect significant heregulin mRNA expression in cancer-associated gastric fibroblasts (Figure S4). This highlights the potential involvement of stromal cells in tumor resistance, as shown here through the expression of heregulin. Many studies on the tumor biology of oncogenic growth factor receptors focus on their expression, basal activity, and downstream signaling in tumor cells. Our findings underline the importance of extending the analyses towards the possible impact of the respective receptor ligands, to better understand and predict the effects of targeted therapeutics in the actual in vivo context. 

## 4. Material and Methods

### 4.1. Materials

Cell culture media, phosphate buffered saline, and fetal bovine serum were obtained from Invitrogen (Gibco, Karlsruhe, Germany). Antibodies against Akt, phospho-Akt, p44/42 MAPK, phospho-p44/42 MAPK, actin, and anti-mouse IgG Alexa Fluor^®^ 647 (#4410S) were purchased from Cell Signaling (Danvers, MA, USA). Anti-HER3 purified antibody (clone 1B4C3) was purchased from BioLegend^®^. Secondary antibodies were from Sigma–Aldrich (Steinheim, Germany). Protran Nitrocellulose Transfer membranes were purchased from Whatman (Dassel, Germany). The enhanced chemiluminescence systems (Super Signal West Femto Maximum Sensitivity Substrate and SuperSignal West Pico Chemiluminescent Substrate) were from Thermo-Scientific (Bonn, Germany). The WST-1 kit was from Roche Applied Science (Mannheim, Germany). The PCK inhibitor bisindolylmaleimide II (BIM II), the MET inhibitor PF04217903, and the HER1 inhibitor AG1478 were from Tocris (Wiesbaden, Germany). The HER2 inhibitor CP724714 was purchased from Selleckchem (Munich, Germany). Heregulin and 12-O-Tetradecanoylphorbol 13-acetate (PKC activator, PMA) were obtained from Sigma–Aldrich (Steinheim, Germany). The human phospho-MAPK Array Kit was from R&D (Minneapolis, MN, USA). All other chemicals used were purchased from Carl Roth (Karlsruhe, Germany) unless indicated otherwise.

### 4.2. Methods

#### 4.2.1. Cell Culture

Human gastric cancer cell lines MKN7, MKN74, MKN45, SNU5, and Hs746T were obtained from the American Type Culture Collection (ATCC, Manassas, VA, USA). Cell line authentication was monitored regularly by genotyping (Genolytic, Leipzig, Germany). MKN cells were cultured in RPMI 1640 medium (Thermo-Fisher, Waltham, MA, USA) supplemented with 10% (*v*/*v*) heat-inactivated fetal bovine serum (FBS). SNU5 cells were cultured in Iscove’s Modified Dulbecco’s Medium plus 20% (*v*/*v*) FBS. The cell line Hs746T was cultivated in Dulbecco’s Modified Eagle’s Medium (4 mM L-glutamine, 4500 mg/L glucose, 1 mM sodium pyruvate, and 1500 mg/L sodium bicarbonate) supplemented with 10% (*v*/*v*) FBS. All media were used without antibiotics and cells were incubated at 37 °C in a humidified atmosphere containing 5% CO_2_ and passaged every 2–3 days.

#### 4.2.2. Cell Transfection and Treatment

siRNAs (see Table S1 for sequences) were purchased from Eurofins MWG Operon (Ebersberg, Germany). In all knockdown experiments, irrelevant siRNAs targeting luciferase (pGL3) were used as negative control. Prior to transfection, cells were seeded in appropriate cell culture plates and maintained overnight under standard conditions. An amount of 10 nM siRNA (50 nm for SNU5, respectively) were transfected using INTERFERin (Polyplus, Illkirch, France), at 1 μL INTERFERin™/pmol siRNA according to the manufacturer’s protocol. For inhibitor and heregulin treatment, the following concentrations were used: 0.2 µM MET inhibitor unless otherwise state and 20 ng/mL heregulin.

#### 4.2.3. WST-1 Assay 

Cell viability was quantified by measuring the metabolically activated formazan dye from the water-soluble tetrazolium salt WST-1 according to the manufacturer’s protocol. Briefly, cells were seeded into 96-well microplates (Sarstedt, Nümbrecht, Germany) at 1000 cells/well and incubated overnight, prior to siRNA transfection or incubation with inhibitors. At the time points indicated, 10 µl WST solution was added to each well, and after incubation at 37 °C for 60 min, absorbance at 450 nm was measured in a PolarSTAR plate reader from BMG (Offenburg, Germany).

#### 4.2.4. Colony Forming Assay

Five × 10^5^ cells growing in normal growth medium in 25 cm^2^ cell culture flasks were treated with the respective agent for 48 h. Afterwards, cells were trypsinized and counted using a hemocytometer. One thousand cells per condition were re-seeded into a 6-well plate and incubated in normal growth medium (without any further treatment) for 7 days. Thereafter, the medium was aspirated. The colonies were gently washed with PBS, and then stained by use of 0.5% (*w*/*v*) methylene blue in a 1:1 mixture (*v*/*v*) of ethanol and water. The colonies were incubated for 15 min with the staining solution, then gently washed with deionized H_2_O and dried at room temperature. Colonies of more than 50 cells were included in the evaluation.

#### 4.2.5. RNA Isolation and RT-qPCR

Total RNA from cells was isolated using the guanidinium thiocyanate–phenol–chloroform extraction procedure (TRI Reagent, Sigma-Aldrich, Taufkirchen, Germany). The first-strand synthesis was carried out using the RevertAid H Minus First Strand cDNA Synthesis Kit from Fermentas (St Leon-Roth, Germany). Products were amplified using specific, intron-spanning primer pairs, with β-actin or RPLP0 serving as loading controls (for primer sequences, see Table S2). Real-time PCR was performed using the Absolute QPCR SYBR Green Mix from Thermo Fisher Scientific (Schwerte, Germany). To this end, 10 pmol of each primer pair and 4 µl from the 1:100 prediluted first-strand synthesis were added to the reaction mixture, and the PCR was carried out in a light cycler apparatus (LightCycler 2.0 System, Roche Applied Science) using the following conditions: 15 min of initial activation at 95 °C, followed by 55 cycles of 10 s at 95 °C, 10 s at 55 °C, and 10 s at 72 °C each. Fluorescence intensities were recorded after the extension step at 72 °C in each cycle. Crossing points were determined by the software, and the relative gene expression was quantified using the formula:

2^(crossing point of β-actin − crossing point of gene of interest)^ × 100 = relative expression of X vs. housekeeper (percentage of reference gene expression).

#### 4.2.6. Immunoblot

For Western blot analysis, cells were seeded in their respective medium at 2 × 10^5^ cells/well into six-well plates. Cells were serum-starved for 18 h, then stimulated as described in the respective figure legends, washed with ice-cold phosphate-buffered saline, and lysed in 250 µl of lysis buffer containing 5 mM EDTA und 1% (*v*/*v*) NP-40 in PBS. Upon determination of protein concentration using DC™ Protein Assay (Bio-Rad Laboratories, Munich, Germany) lysates containing 25 µg of protein were dissolved in loading buffer (125 mM Tris (pH 6.8), 20% glycerol, 4% SDS, 2% β-mercaptoethanol, and 10 µg/mL bromophenol blue). Twenty microliters of lysate/lane was resolved on 9% SDS-polyacrylamid gels and electroblotted onto nitrocellulose membranes using transfer buffer (191 mM glycine, 25 mM Tris, 10% SDS, and 20% methanol). Blots were incubated for 1 h in Rotiblock to saturate non-specific binding sites, washed in Tris-buffered saline with Tween^®^ 20 (TBST), and incubated in a 1:500 dilution of the phospho-specific anti-p44/42 MAPK or anti-Akt mouse monoclonal antibody in 5% milk powder (*w*/*v*) in TBST. An anti-p44/42 MAPK rabbit monoclonal antibody specific for total (phosphorylated and unphosphorylated) MAPK or Akt served for loading controls. Subsequently, blots were incubated with peroxidase-conjugated secondary antibodies (1:5000). Signals were revealed using enhanced chemiluminescence (Super Signal West Femto Maximum Sensitivity Substrate Kit and SuperSignal West Pico Chemiluminescent Substrate), and visualization was carried out using the Chemismart detection system from Peqlab Biotechnologie (Erlangen, Germany). 

For monitoring the expression of a larger set of MAP kinases, the commercially available Human Phospho-MAPK Array Kit (Proteome Profiler™ Array, R&D, Minneapolis, MN, USA) was employed. Lysates were analyzed in the antibody array according to the manufacturer’s protocol and visualized by chemiluminescence as described above. Signal intensities were quantitated using ImageJ and are shown as heat map (heat mapper software; http://www.heatmapper.ca/) and as a bar diagram.

#### 4.2.7. Flow Cytometry

Cells were harvested and washed 2 times with phosphate buffered saline (PBS). Consequently, cells were resuspended in 100 µL staining buffer (0.5% BSA, 0.1% NaN_3_ in PBS) with 0,125 µg HER3-antibody per sample and incubated at 4 °C overnight. Cells were washed 2 times in staining buffer and incubated with anti-mouse Alexa Fluor^®^ 647 antibody for 1 h at room temperature in the dark before FACS analysis was carried out on a Attune^®^ Acoustic Focusing Cytometer using Attune^®^ Cytometric Software.

#### 4.2.8. Spheroid Outgrow and Spheroid Formation

Tumor spheroids were generated by seeding 1000 cells into agarose-coated 96-well plates. Cells were incubated under normal conditions for 96 h and subsequently analyzed (3D growth) or were transferred into normal 12 well microtiter plates for determination of spheroid outgrowth. For this purpose, transferred spheroids were incubated for further 6 days in normal growth medium. Thereafter, the cells were fixated and stained using 0.5 mg/mL methylene blue in 50% (*v*/*v*) water/ethanol to visualize colony spread and formation of distant colonies.

#### 4.2.9. Statistics

All assays were performed independently at least three times unless indicated otherwise, and either one representative experiment or means +/− S.E.M. of multiple experiments are shown. Densitometric analysis of MAPK array was performed using ImageJ software (NIH, Bethesda, MD, USA). Statistical significance of differences in all assays was assessed by ANOVA with Shapiro–Wilk test using SigmaPlot 13, with *, <0.05; **, <0.01, and ***, <0.001.

## Supplementary Materials

The following are available online at https://www.mdpi.com/1422-0067/22/1/82/s1; Figure S1: MET inhibition by treatment of cells with 0.2 µM PF04217903 produced marked antiproliferative effects in MET-amplified (A) SNU5 and (B) Hs746T cells, Figure S2: Absence of inhibitory effects of 2 µM MET inhibitor PF04217903 in (A) MKN74 and (C) MKN7 cells. Similarly, no visible effects occurred upon siRNA-mediated MET knockdown in (B) MKN74 and (D) MKN7 cells, Figure S3: Upregulation of HER3 protein levels 48 h after treatment of MKN45 cells with 0.2 µM PF04217903, as determined by flow cytometry, Figure S4: Colony formation assay with MKN45 cells pretreated for 48 h with vehicle (DMSO), 0.2 µM PF04217903, 20 ng/mL HRG, or PF04217903 plus HRG. After the treatment phase cells 1000 cells of each preparation were seeded (without any further treatment) and colony growth was monitored. Number of colonies is presented (vehicle treated cells = 100%)., Figure S5: Definition of all spots on the phospho-antibody array shown in Figure 4. On the array, dots are always provided in duplicates, Figure S6: SNU5 cells and MKN45 cells were pretreated with PI3K inhibitor LY294002 (10 µM) or MEK inhibitor PD98059 (10 µM) for 24 h, prior to treatment with MET inhibitor PF04217903 (0.2 µM for 48 h) and analysis of HER3 mRNA expression via RT-qPCR. HER3 expression was normalized to reference gene expression (RPLP0) and is given as percentage of HER3 expression in vehicle treated cells, Figure S7: Western blot analyses of AKT and ERK1/2 activation (phosphorylation). MKN45 (MET amplified) or MKN74 (no MET amplification) cells were serum-starved for 18 h and then treated with MET inhibitor PF04217903 (2 µM for 24 h) plus 50 ng/mL EGF, where indicated. Phospho-AKT and Phospho-ERK1/2 are shown as compared to tubulin as loading control. Note that in MKN45 cells EGF stimulation led to reactivation of ERK1/2 phosphorylation in the presence of MET inhibitor as compared to MET inhibitor alone, whereas AKT phosphorylation is only mildly restored. In MKN74 cells, treatment with MET inhibitor did not affect AKT or ERK1/2 phosphorylation upon EGF stimulation, Figure S8: Expression of heregulin-B in cancer-associated fibroblasts as determined by RT-qPCR. In contrast, all investigated gastric cancer cell lines were found negative, Table S1: siRNA sequences used in the present study for RNAi experiments, Table S2: Primer sequences used in the present study for quantitative PCR analyses.

## References

[1] Den Hoed C.M., Kuipers E.J. (2016). Gastric Cancer: How Can We Reduce the Incidence of This Disease?. Curr. Gastroenterol. Rep..

[2] Van Cutsem E., Sagaert X., Topal B., Haustermans K., Prenen H. (2016). Gastric Cancer. Lancet.

[3] Jemal A., Center M.M., DeSantis C., Ward E.M. (2010). Global Patterns of Cancer Incidence and Mortality Rates and Trends. Cancer Epidemiol. Biomarkers Prev..

[4] Torre L.A., Bray F., Siegel R.L., Ferlay J., Lortet-Tieulent J., Jemal A. (2015). Global Cancer Statistics, 2012. CA Cancer J. Clin..

[5] De Mestier L., Lardière-Deguelte S., Volet J., Kianmanesh R., Bouché O. (2016). Recent Insights in the Therapeutic Management of Patients with Gastric Cancer. Dig. Liver Dis..

[6] Shum H., Rajdev L. (2014). Multimodality Management of Resectable Gastric Cancer: A Review. World J. Gastrointest. Oncol..

[7] Obermannová R., Lordick F. (2017). Management of Metastatic Gastric Cancer. Hematol. Oncol. Clin. N. Am..

[8] Lordick F., Janjigian Y.Y. (2016). Clinical Impact of Tumour Biology in the Management of Gastroesophageal Cancer. Nat. Rev. Clin. Oncol..

[9] Bang Y.J., Van Cutsem E., Feyereislova A., Chung H.C., Shen L., Sawaki A., Lordick F., Ohtsu A., Omuro Y., Satoh T. (2010). Trastuzumab in Combination with Chemotherapy versus Chemotherapy Alone for Treatment of HER2-Positive Advanced Gastric or Gastro-Oesophageal Junction Cancer (ToGA): A Phase 3, Open-Label, Randomised Controlled Trial. Lancet.

[10] Lee H.E., Kim M.A., Lee H.S., Jung E.-J., Yang H.-K., Lee B.L., Bang Y.-J., Kim W.H. (2012). MET in Gastric Carcinomas: Comparison between Protein Expression and Gene Copy Number and Impact on Clinical Outcome. Br. J. Cancer.

[11] Liu Y.J., Shen D., Yin X., Gavine P., Zhang T., Su X., Zhan P., Xu Y., Lv J., Qian J. (2014). HER2, MET and FGFR2 Oncogenic Driver Alterations Define Distinct Molecular Segments for Targeted Therapies in Gastric Carcinoma. Br. J. Cancer.

[12] Ma P.C. (2019). (Not Giving up) the Marathon Race of Met Targeting Therapy: Are We There Yet?. Clin. Cancer Res..

[13] Erjala K., Sundvall M., Junttila T.T., Zhang N., Savisalo M., Mali P., Kulmala J., Pulkkinen J., Grenman R., Elenius K. (2006). Signaling via ErbB2 and ErbB3 Associates with Resistance and Epidermal Growth Factor Receptor (EGFR) Amplification with Sensitivity to EGFR Inhibitor Gefitinib in Head and Neck Squamous Cell Carcinoma Cells. Clin. Cancer Res..

[14] Yamaguchi H., Chang S.S., Hsu J.L., Hung M.C. (2014). Signaling Cross-Talk in the Resistance to HER Family Receptor Targeted Therapy. Oncogene.

[15] Bachleitner-Hofmann T., Sun M.Y., Chen C.-T., Tang L., Song L., Zeng Z., Shah M., Christensen J.G., Rosen N., Solit D.B. (2008). HER Kinase Activation Confers Resistance to MET Tyrosine Kinase Inhibition in MET Oncogene-Addicted Gastric Cancer Cells. Mol. Cancer Ther..

[16] Corso S., Ghiso E., Cepero V., Sierra J.R., Migliore C., Bertotti A., Trusolino L., Comoglio P.M., Giordano S. (2010). Activation of HER Family Members in Gastric Carcinoma Cells Mediates Resistance to MET Inhibition. Mol. Cancer.

[17] Yun C., Gang L., Rongmin G., Xu W., Xuezhi M., Huanqiu C. (2015). Essential Role of Her3 in Two Signaling Transduction Patterns: Her2/Her3 and MET/Her3 in Proliferation of Human Gastric Cancer. Mol. Carcinog..

[18] Han H.J., Russo J., Kohwi Y., Kohwi-Shigematsu T. (2008). SATB1 Reprogrammes Gene Expression to Promote Breast Tumour Growth and Metastasis. Nature.

[19] Frömberg A., Rabe M., Aigner A. (2014). Multiple Effects of the Special AT-Rich Binding Protein 1 (SATB1) in Colon Carcinoma. Int. J. Cancer.

[20] Frömberg A., Rabe M., Oppermann H., Gaunitz F., Aigner A. (2017). Analysis of Cellular and Molecular Antitumor Effects upon Inhibition of SATB1 in Glioblastoma Cells. BMC Cancer.

[21] Zhang S., Tong Y.X., Xu X.S., Lin H., Chao T.F. (2017). Prognostic Significance of SATB1 in Gastrointestinal Cancer: A Meta-Analysis and Literature Review. Oncotarget.

[22] Shinomiya N., Chong F.G., Xie Q., Gustafson M., Waters D.J., Zhang Y.W., Vande Woude G.F. (2004). RNA Interference Reveals That Ligand-Independent Met Activity Is Required for Tumor Cell Signaling and Survival. Cancer Res..

[23] Carraway K.L., Cantley L.C. (1994). A Neu Acquaintance for ErbB3 and ErbB4: A Role for Receptor Heterodimerization in Growth Signaling. Cell.

[24] Cui J.J., McTigue M., Nambu M., Tran-Dubé M., Pairish M., Shen H., Jia L., Cheng H., Hoffman J., Le P. (2012). Discovery of a Novel Class of Exquisitely Selective Mesenchymal-Epithelial Transition Factor (c-MET) Protein Kinase Inhibitors and Identification of the Clinical Candidate 2-(4-(1-(Quinolin-6-Ylmethyl)-1H-[1–3]Triazolo[4,5-b]Pyrazin-6-Yl)-1H-Pyraz. J. Med. Chem..

[25] Ocana A., Vera-Badillo F., Seruga B., Templeton A., Pandiella A., Amir E. (2013). HER3 Overexpression and Survival in Solid Tumors: A Meta-Analysis. J. Natl. Cancer Inst..

[26] Shi J., Qu Y., Li X., Sui F., Yao D., Yang Q., Shi B., Ji M., Hou P. (2016). Increased Expression of EHF via Gene Amplification Contributes to the Activation of HER Family Signaling and Associates with Poor Survival in Gastric Cancer. Cell Death Dis..

[27] Chandarlapaty S., Sawai A., Scaltriti M., Rodrik-Outmezguine V., Grbovic-Huezo O., Serra V., Majumder P.K., Baselga J., Rosen N. (2011). AKT Inhibition Relieves Feedback Suppression of Receptor Tyrosine Kinase Expression and Activity. Cancer Cell.

[28] Pavan Kumar P., Purbey P.K., Sinha C.K., Notani D., Limaye A., Jayani R.S., Galande S. (2006). Phosphorylation of SATB1, a Global Gene Regulator, Acts as a Molecular Switch Regulating Its Transcriptional Activity In Vivo. Mol. Cell.

[29] Purbey P.K., Singh S., Notani D., Kumar P.P., Limaye A.S., Galande S. (2009). Acetylation-Dependent Interaction of SATB1 and CtBP1 Mediates Transcriptional Repression by SATB1. Mol. Cell. Biol..

[30] Mir R., J. Pradhan S., Galande S. (2012). Chromatin Organizer SATB1 As a Novel Molecular Target for Cancer Therapy. Curr. Drug Targets.

[31] Frömberg A., Engeland K., Aigner A. (2018). The Special AT-Rich Sequence Binding Protein 1 (SATB1) and Its Role in Solid Tumors. Cancer Lett..

[32] Sun F., Lu X., Li H., Peng Z., Wu K., Wang G., Tong Q. (2012). Special AT-Rich Sequence Binding Protein 1 Regulates the Multidrug Resistance and Invasion of Human Gastric Cancer Cells. Oncol. Lett..

[33] Peng Z., Wang C., Fang E., Lu X., Wang G., Tong Q. (2014). Co-Delivery of Doxorubicin and SATB1 ShRNA by Thermosensitive Magnetic Cationic Liposomes for Gastric Cancer Therapy. PLoS ONE.

[34] Noguchi H., Sakamoto C., Wada K., Akamatsu T., Uchida T., Tatsuguchi A., Matsui H., Fukui H., Fujimori T., Kasuga M. (1999). Expression of Heregulin α, ErbB2, and ErbB3 and Their Influences on Proliferation of Gastric Epithelial Cells. Gastroenterology.

[35] Nagata K., Wada K., Tatsuguchi A., Futagami S., Gudis K., Miyake K., Tsukui T., Sakamoto C. (2006). Heregulin-α and Heregulin-β Expression Is Linked to a COX-2-PGE2 Pathway in Human Gastric Fibroblasts. Am. J. Physiol. Gastrointest. Liver Physiol..

