# Supplementary material for: SATB1-Mediated Upregulation of the Oncogenic Receptor Tyrosine Kinase HER3 Antagonizes MET Inhibition in Gastric Cancer Cells
Source: Int J Mol Sci. 2020 Dec 23;22(1):82. doi: 10.3390/ijms22010082 (PMC7796274; doi:10.3390/ijms22010082)
Supplement: Supplementary file 1 [file ijms-22-00082-s001.pdf]

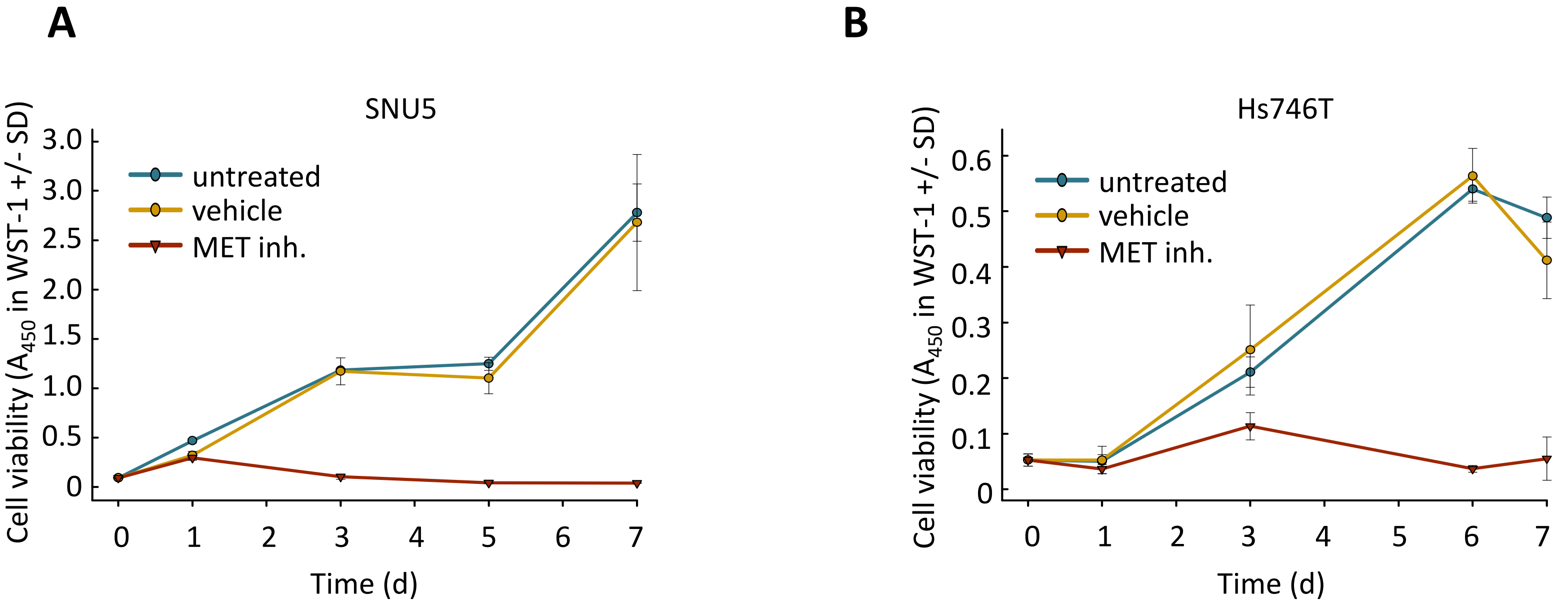

Jenke et al. - Figure S1

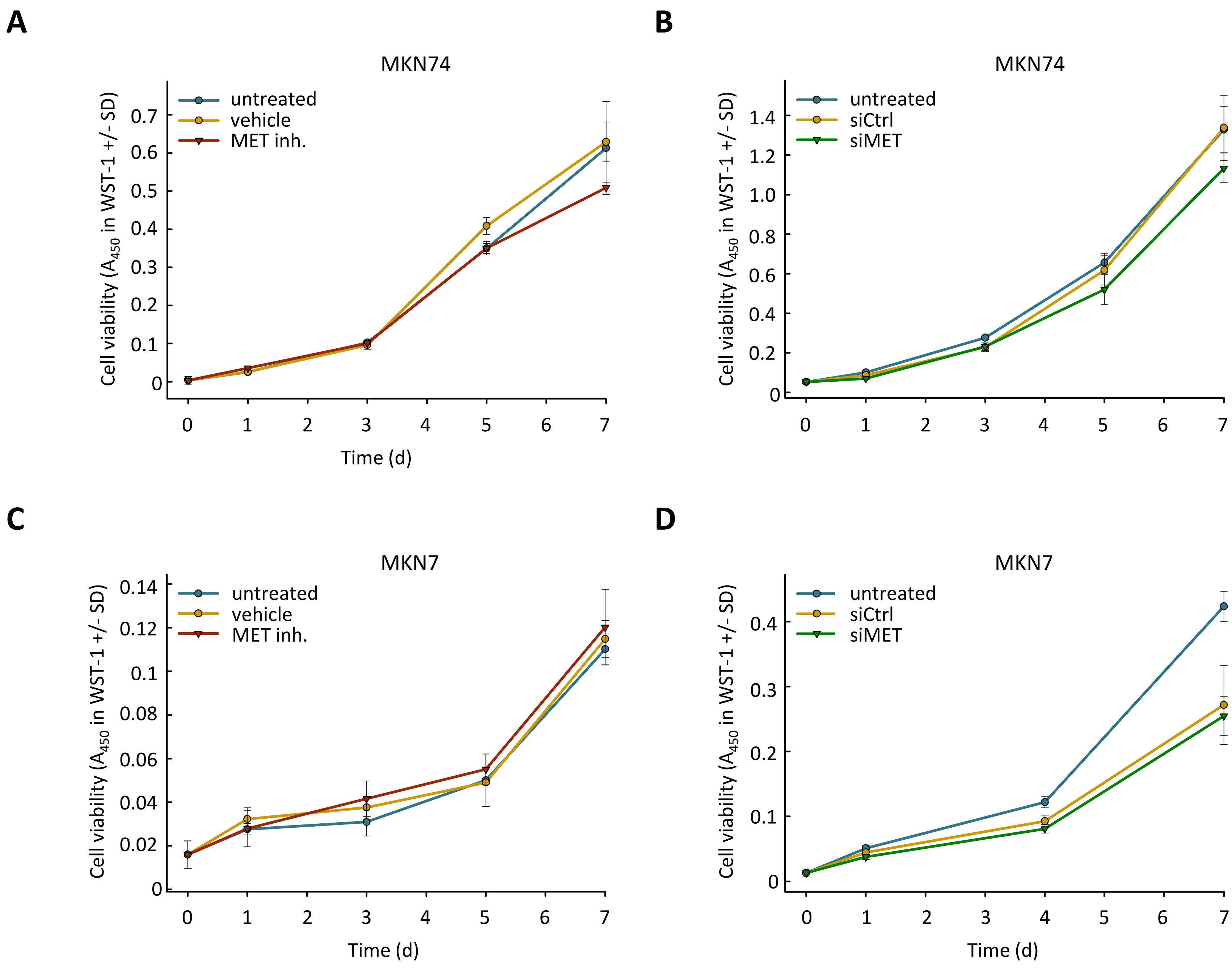

Jenke et al. - Figure S2

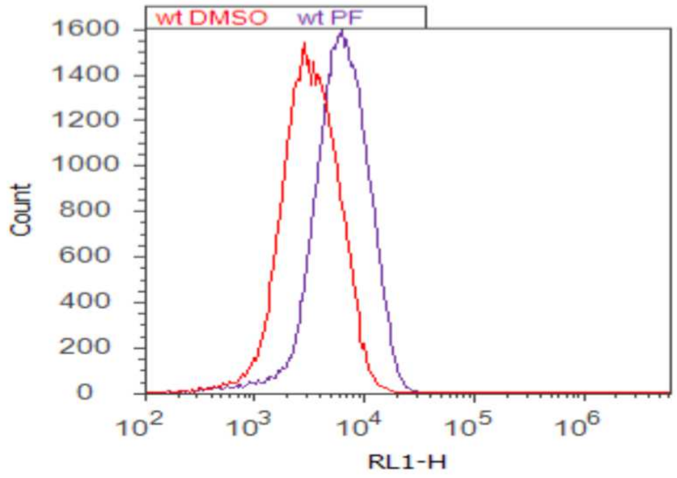

Jenke et al. - Figure S3

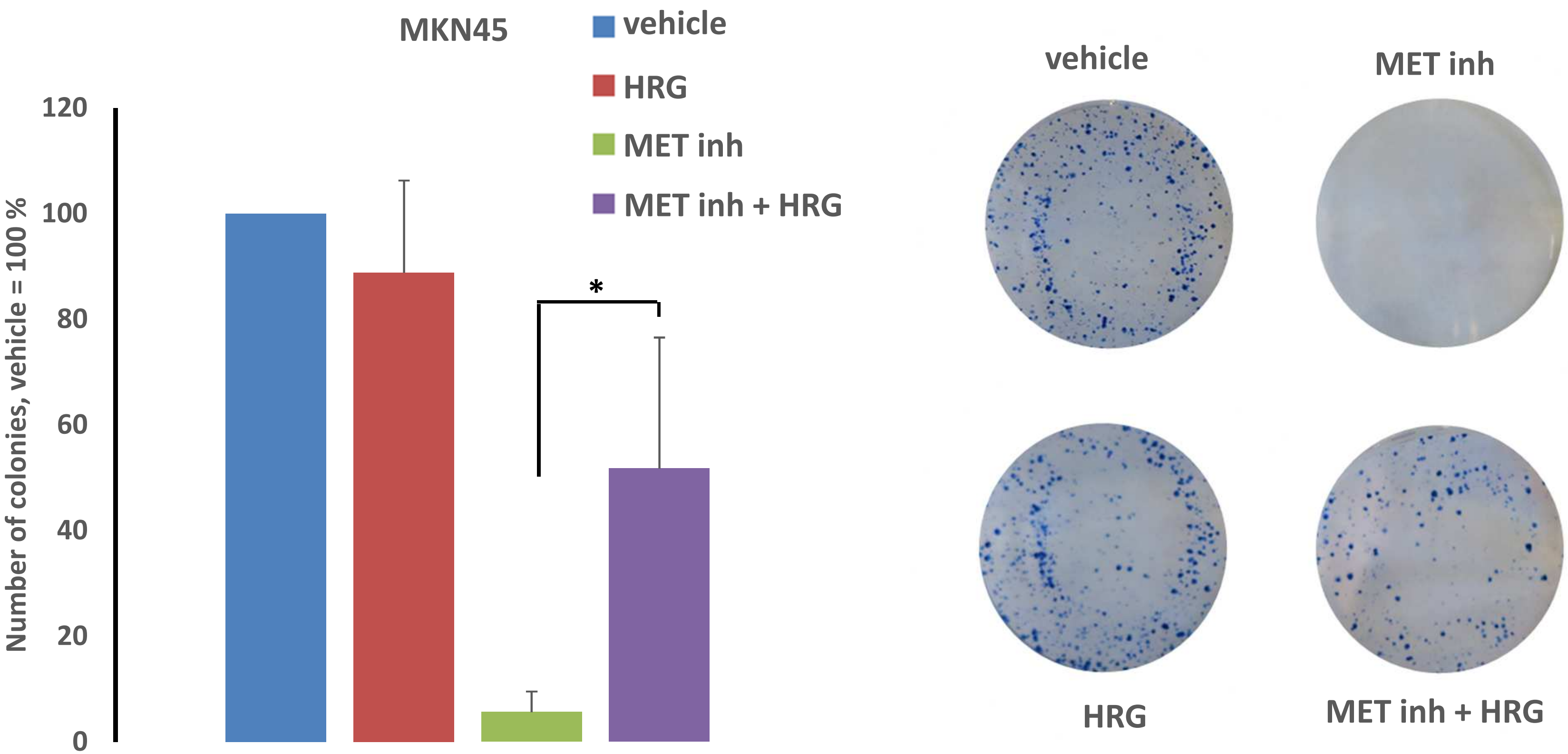

Jenke et al. - Figure S4

Analyte spots

+ control										+ control
	Akt1	Akt2	Akt3	Akt pan	CREB	ERK1	ERK2	GSK3α/β	GSK3β	
	HSP27	JNK1	JNK2	JNK3	JNK pan	MKK3	MKK6	MSK2		
	p38α	p38β	p38d	p38y	p53	p70s6 Kinase	RSK1	RSK2	TOR	
+ control										

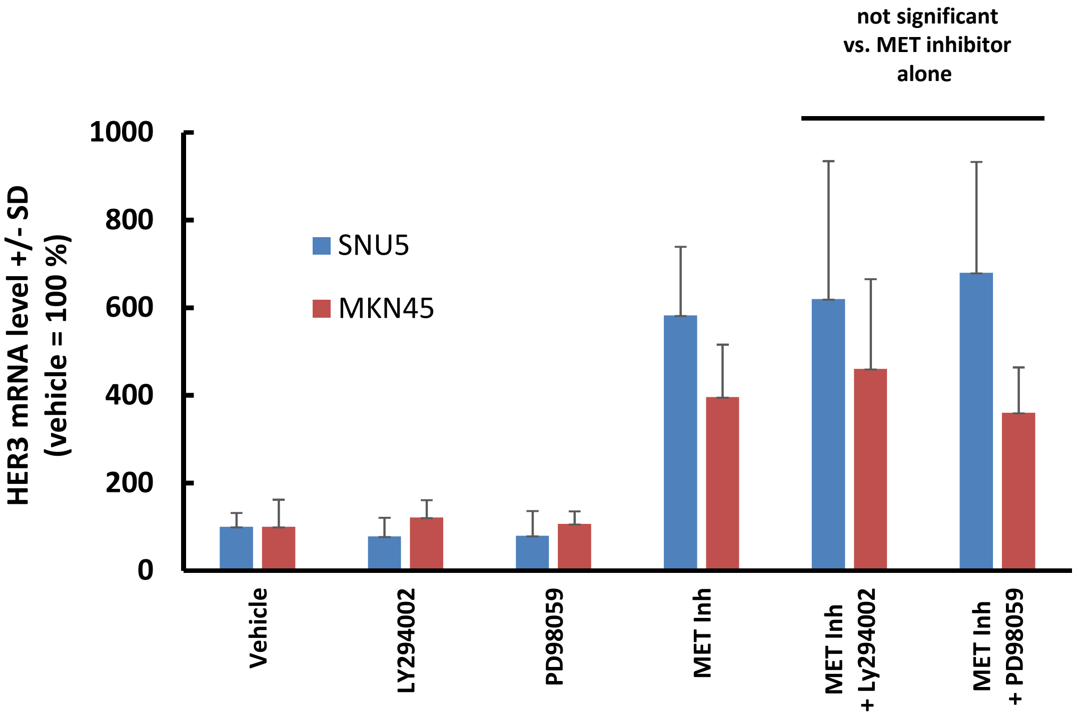

Jenke et al. - Figure S6

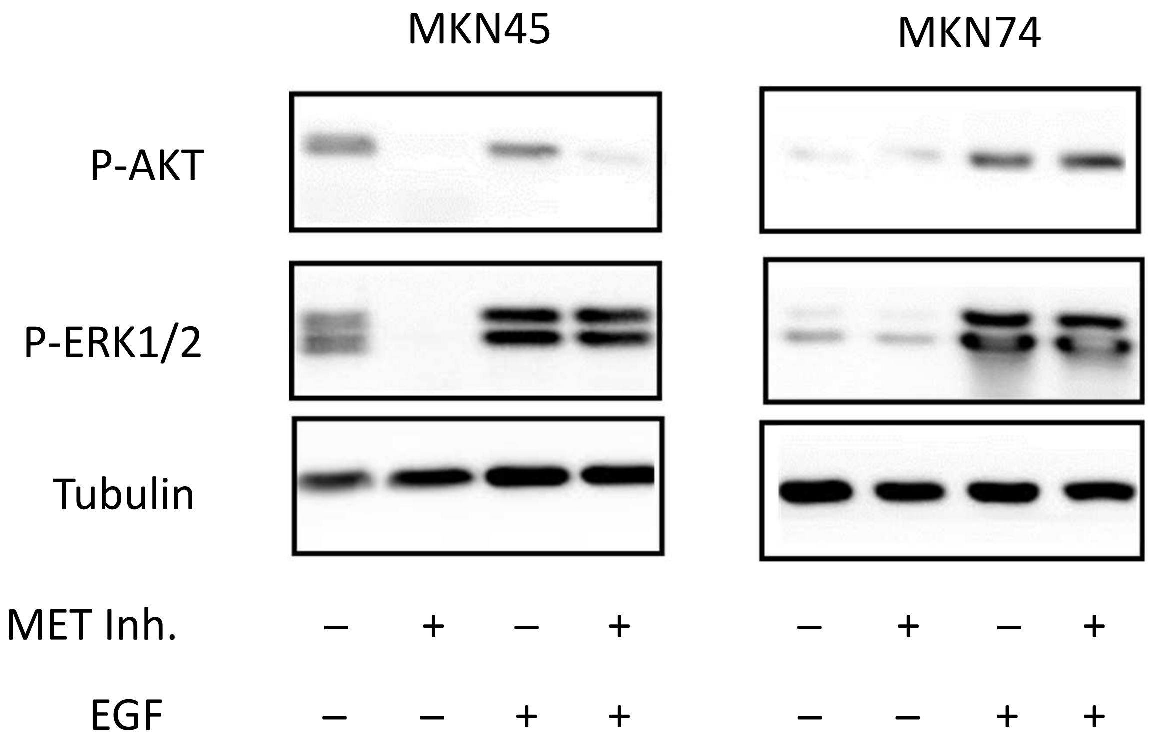
MKN45
MKN74
P-AKT
P-ERK1/2
Tubulin
MET Inh. – + – + – + – +
EGF – – + + – – + +

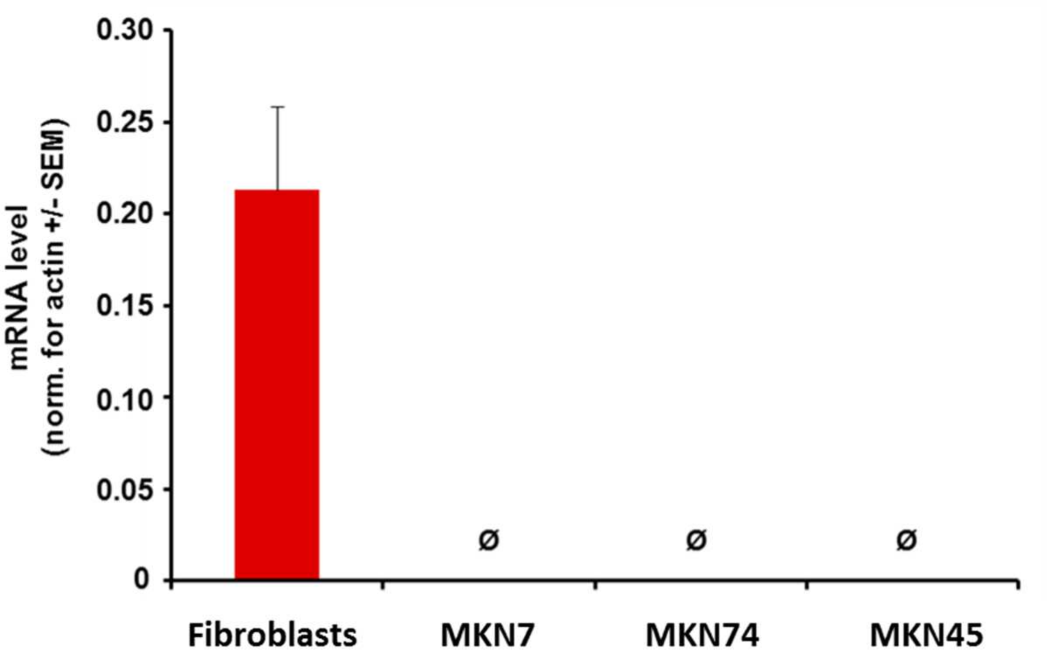

Jenke et al. - Figure S8

Table S1

siRNA sequences used in the present study for RNAi experiments.

Gene	Nucleotide sequence (5’ – 3’)	
HER3	Sense	CCUUGAGAUUGUGCUCACGdTdT
	Antisense	CGUGAGCACAAUCUCAAGGdTdT
MET	Sense	ACUCUAGAUGCUCAGACUUUUUU
	Antisense	AAAAGUCUGAGCAUCUAGAGUUU
SATB1	Sense	GCUUCAAGAUGUGUAUCAUdTdT
	Antisense	AUGAUACACAUCUUGAAGCdTdT

Table S2

Primer sequences used in the present study for quantitative PCR analyses.

**Gene**	**Nucleotide sequence (5' – 3')**	
Actin	Forward	CCAACCGCGAGAAGATGA
	Reverse	CCAGAGGCGTACAGGGATAG
RPLP0	Forward	TCTACAACCCTGAAGTGCTTGAT
	Reverse	CAATCTGCAGACAGACACTGG
HER1	Forward	ACACAGAATCTATACCCACCAGAGT
	Reverse	ATCAACTCCCAAACGGTCAC
HER2	Forward	TGGCTCAGTGACCTGTTTTG
	Reverse	GGTCCTTATAGTGGGCACAGG
HER3	Forward	CTGATCACCGGCCTCAAT
	Reverse	GGAAGACATTGAGCTTCTCTGG
MET	Forward	AAATGTGCATGAAGCAGGAA
	Reverse	TCTCTGAATTAGAGCGATGTTGA
SATB1	Forward	CGATGAACTGAAACGAGCAG
	Reverse	CGGAGGATTTCTGAAAGCAA
